# Comparing different nonlinearities in readout systems for optical neuromorphic computing networks

**DOI:** 10.1038/s41598-021-03594-0

**Published:** 2021-12-17

**Authors:** Chonghuai Ma, Joris Lambrecht, Floris Laporte, Xin Yin, Joni Dambre, Peter Bienstman

**Affiliations:** 1 Photonics Research Group, Ghent University - imec, Ghent, 9052 Belgium; 2 IDLab, Ghent University - imec, Ghent, 9052 Belgium

**Keywords:** Silicon photonics, Computer science

## Abstract

Nonlinear activation is a crucial building block of most machine-learning systems. However, unlike in the digital electrical domain, applying a saturating nonlinear function in a neural network in the analog optical domain is not as easy, especially in integrated systems. In this paper, we first investigate in detail the photodetector nonlinearity in two main readout schemes: electrical readout and optical readout. On a 3-bit-delayed XOR task, we show that optical readout trained with backpropagation gives the best performance. Furthermore, we propose an additional saturating nonlinearity coming from a deliberately non-ideal voltage amplifier after the detector. Compared to an all-optical nonlinearity, these two kinds of nonlinearities are extremely easy to obtain at no additional cost, since photodiodes and voltage amplifiers are present in any system. Moreover, not having to design ideal linear amplifiers could relax their design requirements. We show through simulation that for long-distance nonlinear fiber distortion compensation, using only the photodiode nonlinearity in an optical readout delivers BER improvements over three orders of magnitude. Combined with the amplifier saturation nonlinearity, we obtain another three orders of magnitude improvement of the BER.

## Introduction

Machine learning is getting more and more ubiquitous in everyday life. With increasing demands from all kinds of applications, the vast majority of machine-learning hardware is based on von-Neumann architectures, like CPUs and GPUs. While these are compelling and capable of high-complexity computations, they are inherently limited in power efficiency and latency because of their digital electronic nature. This is especially the case in digital signal processing for the optical communication industry, where the signal bandwidth has been getting higher and higher in recent years. With a tens-of-gigabaud transmission data rate, real-time signal processing in electronic digital signal processors (DSPs) is getting progressively harder to achieve, at an increasing cost in terms of energy, latency and chip area^1^.

For this reason, it is interesting to also consider other information processing paradigms, like those based on neuromorphic computation. In addition, optical neuromorphic networks have the added advantage of being able to operate in the optical domain, promising much better processing speeds and power efficiency^2,3^. All-optical neuromorphic systems are especially useful for optical communication systems, since they do not require optical-electrical conversion of the signal within the network, which translates to additional energy savings. Instead, the optical signal can be transported and manipulated directly in the optical domain by photonics hardware and only be detected at the end of the system. Apart from all-optical neuromorphic networks, there are also systems that implement hybrid neural networks^4^, consisting of both optical and electrical components, as well as neural networks based on diffractive elements^5^.

Photonics reservoir computing^6–8^ is one of the hardware implementations of optical neuromorphic processing. The scheme of reservoir computing (RC) was introduced two decades ago^9,10^ and consists essentially of a recurrent neural network (RNN), except for the fact that the weights of the internal nodes are not trained. The training of an RC network only requires the optimization of a linear readout layer, which makes it much easier than training a conventional RNN system. Moreover, the internal connection weights can be set randomly without further constraints. This feature makes the hardware manufacturing of such a system more robust than others, as deviations due to fabrication tolerances can be compensated for by having bespoke weights for each chip. Additionally, like RNNs, RC systems are inherently suited to the processing of time-varying signals, because of the presence of recurrent connection between their nodes which gives them internal memory.

*Nonlinearity* Nonlinearity is one of the essential building blocks of a machine learning network. In the digital electrical domain, an arbitrary nonlinear function is relatively easy to implement. However, in the analog optical domain, performing a nonlinear activation function on an analog optical signal brings many challenges. There are some approaches that utilize nonlinearity in the optical domain, like the nonlinearity based on optical reverse saturated absorption^11^, or utilizing integrated semiconductor optical amplifiers^6^. However, such optical devices often require high external optical or electrical power and are usually not cost-efficient or difficult to integrate on-chip. Therefore, many papers consider a hybrid optical neural network, where the nonlinearity is achieved in the electrical domain^12^ as a workaround. However, this sacrifices latency and power efficiency because of the process of opto-electrical and electro-optical conversion. Another option is external electrical-modulation-assisted nonlinearity^13^, which utilizes a special driving of optical modulators. However, such an implementation would also introduce additional complexity to the system. In short, the methods mentioned above normally require a complex setup or high optical or electrical power.

Another approach does not consider any nonlinearity *inside* the network, but rather only relies on the detector nonlinearity at the *output* of the system^7,14^. In that case, the nonlinearity mainly comes from the modulus-square of the optical detection from the photodetector. As mentioned before, this nonlinearity is easy to obtain at no extra cost. However, as we will discuss in this paper, there are some pitfalls to be aware of when exploiting this non-linearity, especially if the weighted sum happens before the detector in the optical domain.

The goal of this work is to demonstrate the possible performance improvements by fully utilizing the nonlinearities from the photodetector. We also propose to use another easily accessible nonlinearity, namely that of a deliberately non-ideal voltage amplifier that is part of the transimpedance amplifier (TIA) module present in any readout system. These combined nonlinearities deliver high performance while still being very easy to realise experimentally. Additionally, we slightly ease the design of a traditional voltage amplifier, since we no longer require a stringent linear behaviour from its emitter-coupled pair.

The rest of this paper is structured as follows. In “Reservoir computing architecture” section, we introduce the reservoir computing system we used for the simulations in the paper. In “Photodetector nonlinearity: electrical readout versus optical readout” section we compare so-called electrical and optical readout schemes and show their performance difference when utilizing the photodetector nonlinearity. “Voltage amplifier nonlinearity” section deals with the improvement coming from exploiting nonlinearity in voltage amplifiers, in addition to the detector nonlinearity. We conclude our work in “Conclusion” section.

## Reservoir computing architecture

In principle, the optical input to the readout system can be the output of the final layer of any photonic neuromorphic architecture, as long as that signal is coherent. The goal of our work is not to compare different photonic neuromorphic architectures, which each have their strengths and weaknesses depending on the application. Rather, we focus on the readout part at the end of the system, which performs linear combinations and a non-linear transformation. In this paper, we illustrate the influence of this readout layer using a neural architecture called reservoir computing (RC).

As mentioned above, reservoir computing consists of an untrained recurrent neural network (the reservoir) and a trained linear readout layer.

In discretized time, a general form of the reservoir state update equation is given by:

$$\begin{aligned} {x}[k+1] = f \left( {W}_{res}{x}[k] + {w}_{in}({u}[k+1] + {u}_{bias}) \right) \end{aligned}$$

Here *f* is a nonlinear function, *u* is the input to the reservoir and ${u}_{bias}$ is a fixed scalar bias applied to the inputs of the reservoir. For an *N*-node reservoir, ${W_{res}}$ is an $N \times N$ matrix representing the interconnections between reservoir components. ${w}_{in}$ is an *N*-dimensional column vector whose elements are nonzero for each active input node.

The output *y* is given by a simple linear combination of the states:

$$\begin{aligned} y[k] = {W}_{out} {x}[k] \end{aligned}$$

In terms of photonic implementations, there are two main classes. The first one is based on time-multiplexed systems that utilize a single nonlinear element which is coupled to a feedback loop with a curated delay time^15–18^. The second approach consists of spatially extended systems, where each node corresponds to a separate device, e.g. a network of optical amplifiers^19^. A variation on this are optical networks which do not contain any nonlinearity inside the network, but rely on the detector nonlinearity at the output and are therefore purely passive^6,20,21^.

**Figure 1:**
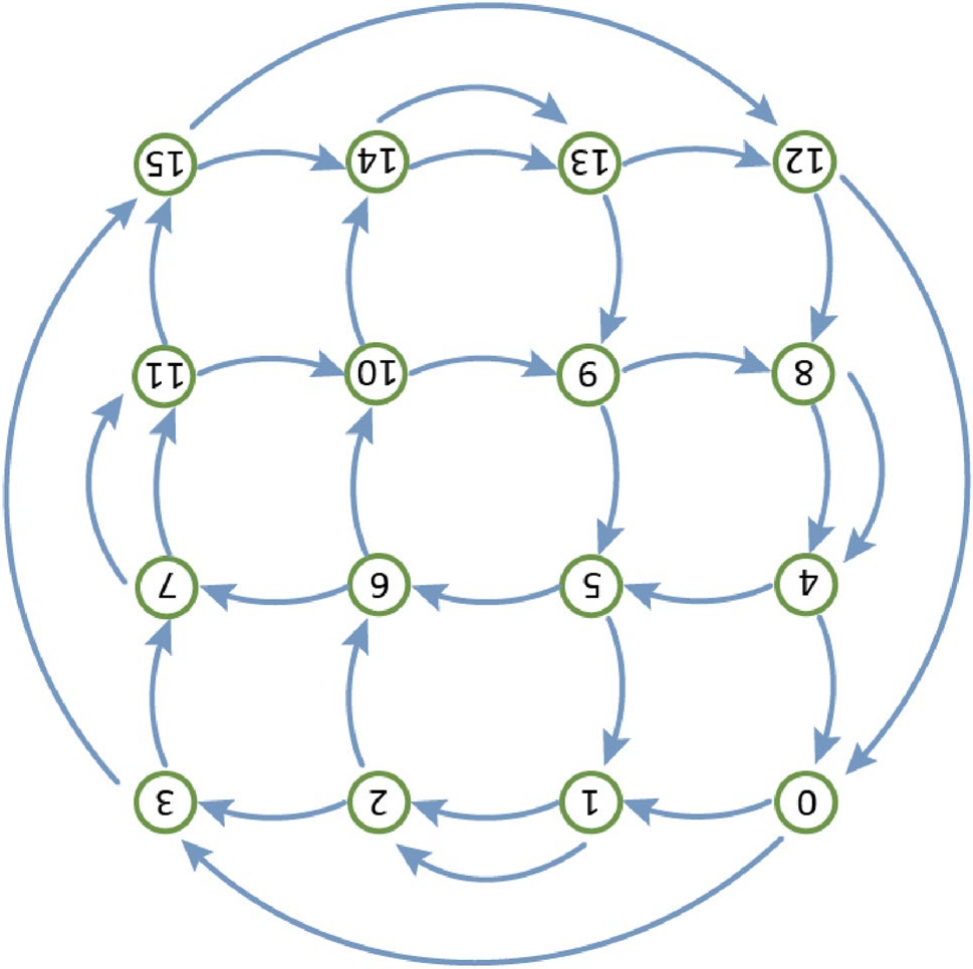
4-port topology that we used as our reservoir architecture. Shown here is a 16 node layout. Each node has two inputs and two outputs that are connected to other nodes.

In the simulations for this paper, we make use of a 16-node integrated silicon RC computing system, which uses 50/50 directional coupler pairs as nodes, which are interconnected using the so-called 4-port topology^20^ that is illustrated by Fig. 1, mainly to reduce the optical loss in the reservoir.

We use the Photontorch^22^ framework for circuit simulation, which is a highly configurable parallel photonics circuit simulation and optimization framework based on Pytorch tensors^23^. This also allows easy interfacing with machine-learning optimization techniques.

## Photodetector nonlinearity: electrical readout versus optical readout

### Readout systems

A readout system takes the output of the optical neuromorphic network as its input, applies some signal processing and produces a final electrical signal as output. The signal processing usually consists of weighting and summation of the inputs, and can include additional nonlinearity.

**Figure 2:**
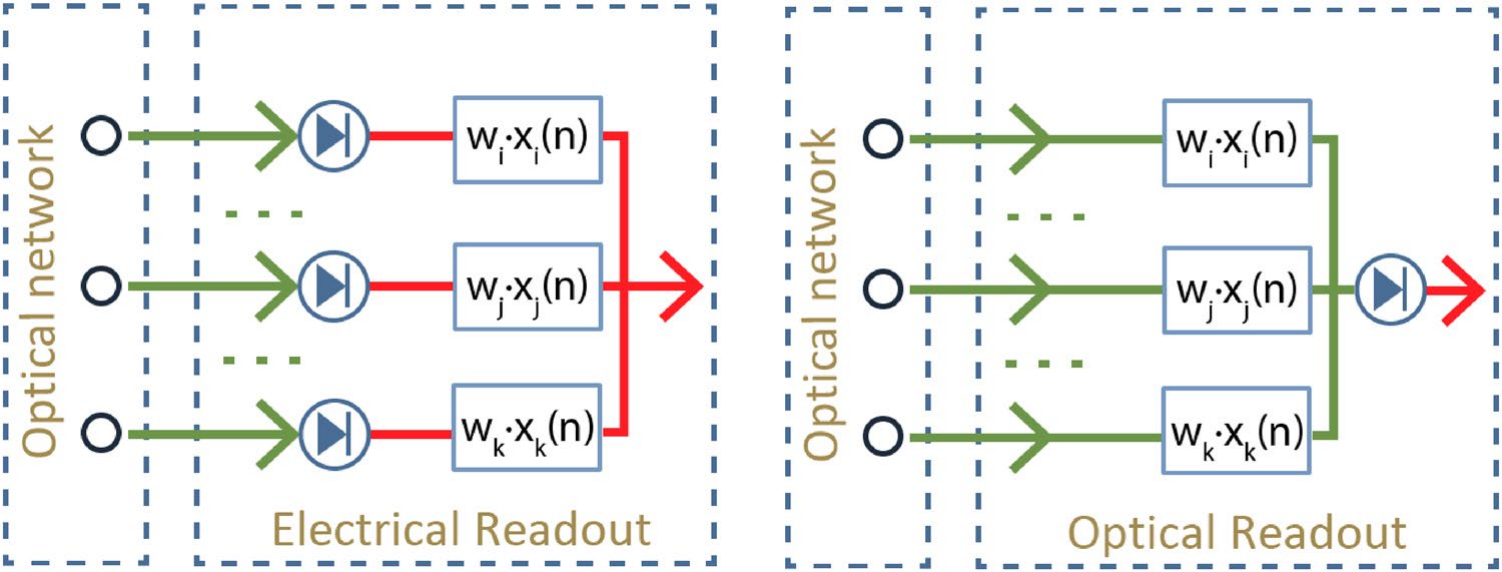
(Left) electrical readout. (Right) optical readout. Green lines represent optical signals, and red lines electrical signals. A crucial difference is the order of the summation and detection.

We distinguish two readout schemes. In a so-called electrical readout scheme (Fig. 2 left), optical detection is first performed for each individual input node. This is then followed by weighting in the (analog or digital) electrical domain. Note that this scheme requires a separate detector and analog-to-digital converter (ADC) for each node, which is not desirable.

On the other hand, in a so-called optical readout scheme (Fig. 2 right), the weighting is performed in the analog optical domain, e.g. using phase shifters or modulators, and then the weighted signals are coherently combined in an optical combiner tree. In this scheme, only a single detector/ADC is needed.

Another conceptual difference between these two schemes is the order of the weighting, summation and (nonlinear) detection. One can indeed expect that the richness that can be achieved by the nonlinearity will depend on whether or not the summation happens before it. Before comparing the performance of these two readouts, we will first discuss the consequences of this ordering on the way these systems are trained, i.e. on the way the weights in the linear combination should be chosen in order to minimise the difference between the desired output and the actual output.

### Training the electrical readout

In this scheme, after applying the inherent nonlinear transformation in the photodiode (from complex-valued fields to real-valued intensities), we need to find a set of real-valued weights to sum these time traces in order to approximate the target time trace. This is essentially a linear regression operation:

$$\begin{aligned} W_{out} X = \tilde{Y} \end{aligned}$$

Here *X* is the matrix containing the input time traces for each node, $\tilde{Y}$ is the desired target signal, and $W_{out}$ are the output weights that need to be found so as to minimise the difference between $W_{out} X$ and $\tilde{Y}$. This can be achieved in one step using the Moore-Penrose generalized matrix inverse^24^, where a regularization parameter $\lambda$ can also be taken into consideration:

$$\begin{aligned} W_{out} = (X^TX + \lambda I)^{-1}X^T\tilde{Y} \end{aligned}$$

This one-step solution vastly reduces the optimization time and makes training much more accessible, as we do not have to perform iterations which could get stuck in local minima. This regression is the standard method for reservoir computing training when the weighting is done in the electrical domain.

### Training the optical readout

Mathematically, when the signal is in the optical domain and is coherent (i.e. complex valued), a similar linear regression one-step solution still exists, which is:

$$\begin{aligned} W_{out} = (X^HX + \lambda I)^{-1}X^H\tilde{Y} \end{aligned}$$

Here, the signals are represented by a complex-valued matrix and the conjugate transpose is used instead of regular transpose.

In principle this solution can be used to train coherent optical readout systems. However, the issue is choosing the complex-valued target. Indeed, for many applications, the only target we care about is a real-valued target after the photodiode, and not a complex-valued target before that same photodiode. Since in that case the phase before the detector is irrelevant, we could in theory fix it arbitrarily to e.g. zero and then perform the complex-valued linear regression using that target. However, in that way we artificially restrict our solution space to situations where the phase before the detector is zero. As such, we risk missing out on better solutions.

Therefore, a better approach is to abandon linear regression, and to use backpropagation to train the entire system, including the nonlinearity in the readout^22^. That way, we only need to specify the real-valued target after the detector, and the phase before the detector is free to evolve as the optimiser sees fit. Therefore, unnecessary constraints and assumptions are eliminated.

In our simulations we use Pytorch^23^ as the backpropagation optimization framework. The optimizer of choice is Adam^25^. Since the number of nodes is not significant and the readout system itself only consists of one layer of weighting, the optimization time is usually below 1 minute, with GPU acceleration enabled by an NVIDIA Geforce 970 graphics card.

### Benchmark: 3-bit-delayed XOR task

**Figure 3:**
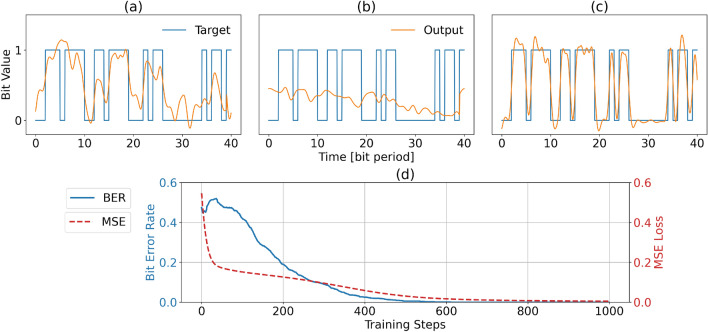
Performance comparison for the 3-bit delayed XOR task, for electrical (**a**) and optical (**b**,**c**) readouts. In (**b**), the readout is trained by linear regression and in (**c**) backpropagation through the photodetector is performed. It is clear that for this last option the actual output of the system (orange) most closely resembles the target (blue). (**d**) Evolution of Bit Error Rate and Normalised Mean Square Error during the backpropagation training.

In this section, we show the performance of the two readout schemes on the 3-bit delayed XOR task. Among the basic logic gates, a boolean XOR requires the most nonlinearity in the calculation. Here, we choose a more challenging variant of this task, namely the 3-bit-delayed XOR, which calculates the XOR between the current bit and the one from 3 bit periods ago. As such, it also requires more memory from the photonics system. To train the system, we use 2000 randomly generated bits and another 2000 bits as testing data. The simulated bit rate is 32 Gbit/s and the CW laser works at 1550 nm wavelength with operation power of 5 mW. For the photodetector, we modeled a PIN photodiode based on the model from VPI Design Suite simulation framework that takes dark current, shot noise and thermal noise into account^26–29^ The PIN photodetector has a responsivity of 1, dark current of $1e^{-10}$ A, a load resistance of 166 Ohm. The bandwidth of the detector used in this task is 16 GHz, which is modeled by a Butterworth filter of 4th order. The same detector model is used for our next benchmark task. The performance comparison and training progress are illustrated in Fig. 3.

For this nonlinear task with memory, Fig. 3a shows that, although there is nonlinearity involved in the electrical readout, it still does not deliver good enough performance. Figure 3b shows that the complex regression, which ignores the photodetector in the optimization, does not perform well at all. The nonlinearity of the detector still exists, but it only applies a square nonlinearity on the single optical output signal converting it to electrical power. The phase of the optical signal before the detector is artificially constrained, limiting the system in giving a valid prediction. In Fig. 3c, we do include the photodetector nonlinearity in the optimization, resulting in a very clean signal that follows the target signal very well, with a practically zero bit error rate.

## Voltage amplifier nonlinearity

### Voltage amplifiers

The idealised photodiode discussed in the previous section only provides a square nonlinearity. However, machine learning teaches us that saturating nonlinearities are very important to implement as well. As discussed in the introduction, obtaining such nonlinearities in an optical neural network often requires either certain optical nonlinear effects to emulate the saturation nonlinearity, or external electrical assistance. In comparison, exploiting the saturating nonlinearity in the voltage amplifier in a TIA module is much easier to obtain, and the profile of the nonlinearity is essentially a hyperbolic tangent function ($\tanh$).

Indeed, for an emitter-coupled amplifier pair. the gain is already nonlinear. The differential output voltage $V_{od}$ as a function of the differential input voltage $V_{id}$ is given by^30^:

$$\begin{aligned} V_{od} = \alpha _FI_{\mathrm {TAIL}}R_C \tanh (-V_{id}/2V_T) \end{aligned}$$

**Figure 4:**
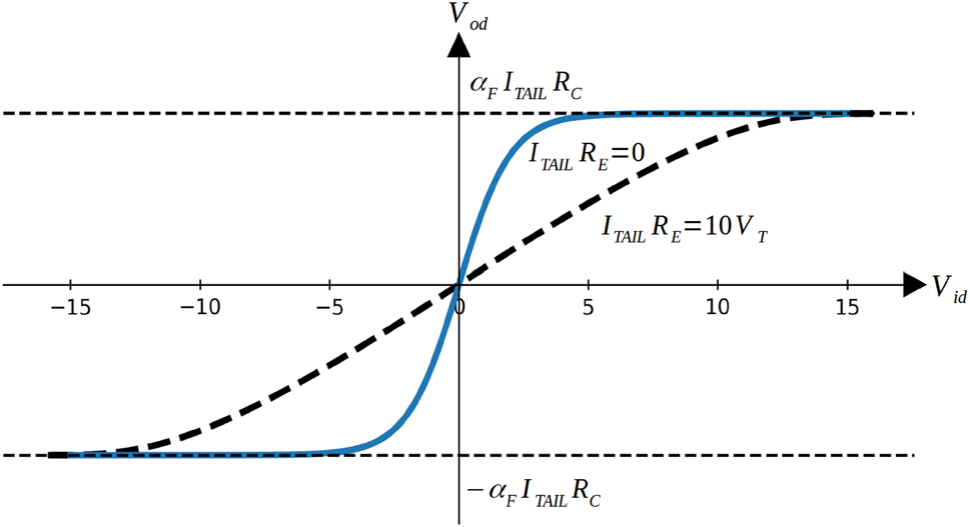
Voltage amplifier gain curve. The black dashed curve represents a regular gain curve obtained by using emitter-degeneration resistors ${R_E}$. The blue curve represents the initial gain curve without an emitter-degeneration resistor.

Here, $\alpha _F$ represents the common-base current gain and should be very close to 1. $I_\mathrm {TAIL}$ and $R_C$ are respectively the bias current and the load resistor in the amplifier. $V_T$ is the threshold voltage, which is usually determined by the operation temperature. As mentioned before, the input and the output have a nonlinear relation given by the $\tanh$ function. In common amplifier design, it is important to avoid the nonlinear range of $V_{id}$. To achieve that, emitter-degeneration resistors $R_E$ are normally added to enlarge the linear region of the amplification curve. The transmission relation is then shown below in Fig. 4. By introducing large emitter resistance (see Fig. 4 black dashed curve, which corresponds to an emitter-degeneration resistor of $R_E = 10V_T/I_\mathrm {TAIL}$ ), the linear range will be extended considerably, but at the expense of a lower voltage gain.

However, in order to exploit the amplifier nonlinearity for machine-learning purposes, one can lower the emitter resistance to make the gain more nonlinear, or even remove the resistor entirely to obtain a pure hyperbolic tangent gain curve. Potentially, the strength of the nonlinearity can even be predesigned or even tuned during testing: with $R_E$ present and fixed, the linear range is determined by $I_\mathrm {TAIL}R_E$. So increasing $I_\mathrm {TAIL}$ then gives a more linear range and less voltage gain. Additionally, tuning $R_E$ on-chip is not difficult either.

### Benchmark: long-distance nonlinear fiber distortion compensation

To demonstrate the performance benefit of utilizing this nonlinearity, we choose to perform long-haul nonlinear fiber distortion compensation. This task is both harder and more industrially relevant than the XOR task. Fiber dispersion and nonlinear distortion are significant factors that degrade the optical signal quality in the fiber^27^. Linear dispersion from the fiber is comparatively easy to compensate in terms of computational capability. However, nonlinear distortion (especially for long-distance communication) requires better computational capability when doing the signal equalization. Modern solutions like digital backpropagation^31^ and Volterra series^32^ typically require extensive DSP (digital signal processing)^33^ for such tasks after the optical signal is detected and digitized by analog-to-digital converters (ADCs). An all-optical system without any optical-electrical conversion in between could bring benefits in minimizing latency and power consumption.

In our simulation, we obtain the distorted optical signal data from the optical system simulation framework VPIphotonics Design $\hbox {Suite}^{TM}$^26^. The transmission system we choose is a 2000 km NRZ transmission link. The transmission speed is 10 Gbit/s. Detailed parameters of the system are listed in Table 1. The link consists of ten repetitive sections of 200 km fiber span. In each section there are regular single-mode fibers with dispersion compensation modules (DCM) for managing linear dispersion, and two erbium-doped fiber amplifiers (EDFAs), one at the beginning of the link as booster and a second one before the dispersion compensation fiber as line amplifier for loss management. In this case, the linear dispersion of the link is already mitigated and the final optical signal distortion is dominated by the (harder to compensate) nonlinear effects.

**Table 1:** Details of the long distance transmission link.

Link details	
Parameter name	Value
Laser power	2 mW
Laser wavelength	1550 nm
Laser linewidth	10 MHz
Transmission bit rate	10 Gbit/s
Laser emission frequency	$193.1\times 10^{12}$ Hz
Transmission fiber dispersion	$16\times 10^{-6}\,\text {s}/\text {m}^2$
Transmission fiber nonlinear index	$5.6\times 10^{-20}\,\text {m}^2/\text {W}$
DCM fiber dispersion	$106\times 10^{-6}\,\text {s}/\text {m}^2$
DCM fiber nonlinear index	$4.4\times 10^{-20}\,\text {m}^2/\text {W}$
Detector bandwidth	10 GHz

Training details	
Parameter name	Value
Training set size	2048 bit
Testing set size	4096 bit
Loss function	Mean square error
Optimizer	Adam

**Figure 5:**
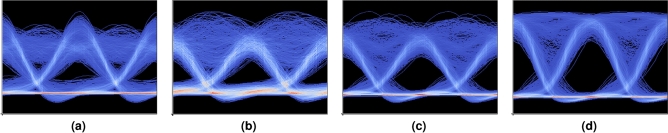
Eye diagrams for different readout schemes and with different nonlinearities. (**a**) Unequalized, (**b**) equalized by electrical readout, (**c**) equalized by optical readout and (**d**) equalized by optical readout utilizing both photodetector nonlinearity and voltage amplifier nonlinearity.

The results from Fig. 5 show the performance for this nonlinear distortion compensation task in the form of eye diagrams. It includes all the previously discussed readout schemes and nonlinearities, so as to compare their performance differences.

By performing a Gaussian distribution bit-error-rate (BER) approximation method with Inter-Symbol-Interference (ISI) taken into account^34^, we can statistically estimate the BER to levels much lower than what would be possible with simple error counting. Using this method, the BER from the original unequalized signal (Fig. 5a) can be calculated to be around $10^{-7}$. Using an electrical readout, the system can slightly improve the BER with one order of magnitude to $10^{-8}$, as shown in Fig. 5b. The result is also in alignment with the observation we got from the XOR task before, where electrical readout performed reasonably well. Even though the electrical readout scheme incorporates the photodetector nonlinearity, without utilizing coherent weighting, its performance is still limited. In comparison, the eye diagram of the optical readout system trained with backpropagation is more open both in the vertical and the horizontal direction (Fig. 5c), with a BER of $10^{-10}$. This again proves that the optical readout system can utilize the nonlinearity in the photodetector better and provide enough computational capability to compensate for the nonlinear distortion. Finally, combined with the voltage amplifier nonlinearity, the BER will have a further improvement to $10^{-13}$ (Fig. 5d). We note here that the nonlinearity required by the amplifier is not extreme. The current nonlinear region we utilize in our system is only about 55% of the 3 dB compression point (P3dB).

We need to stress that the compensation here is done purely in the optical domain, before the detection by the photodetector. This analog optical dispersion compensation has the potential to reduce the requirements on the DSP after the detection.

## Conclusion

In this work, we discussed two types of readout systems for coherent neuromorphic computing networks using the easily accessible photodetector nonlinearity. We also demonstrated a further performance boost by exploiting the gain nonlinearity from the voltage amplifier in the detection link. In our work, we used a basic 16-node reservoir computing network without any form of nonlinearity inside as the input to the readout system. By comparing electrical readout and optical readout systems on the 3-bit delay XOR task, we demonstrated that by performing the proper training method, the optical readout can utilize the photodetector nonlinearity much better than the electrical readout. For a long-distance and harder nonlinear fiber distortion compensation task, we further demonstrate that combining the photodetector nonlinearity with amplifier nonlinearity results in a system that is capable of delivering a six orders of magnitude BER improvement.

## References

[1] Kuschnerov M (2009). DSP for coherent single-carrier receivers. J. Lightwave Technol..

[2] Lugnan A (2020). Photonic neuromorphic information processing and reservoir computing. APL Photo..

[3] Shastri, B. J. *et al.**Photonics for artificial intelligence and neuromorphic computing*. arXiv preprint arXiv:2011.00111 (2020).

[4] Shen Y (2017). Deep learning with coherent nanophotonic circuits. Nat. Photo..

[5] Lin X (2018). All-optical machine learning using diffractive deep neural networks. Science (New York, N.Y.).

[6] Vandoorne K (2014). Experimental demonstration of reservoir computing on a silicon photonics chip. Nat. Commun..

[7] Katumba A, Yin X, Dambre J, Bienstman P (2019). A neuromorphic silicon photonics nonlinear equalizer for optical communications with intensity modulation and direct detection. J. Lightwave Technol..

[8] Van der Sande G, Brunner D, Soriano MC (2017). Advances in photonic reservoir computing. Nanophotonics.

[9] Jaeger H, Haas H (2004). Harnessing nonlinearity: Predicting chaotic systems and saving energy in wireless communication. Science.

[10] Maass W, Natschläger T, Markram H (2002). Real-time computing without stable states: A new framework for neural computation based on perturbations. Neural Comput..

[11] Miscuglio M (2018). All-optical nonlinear activation function for photonic neural networks. Opt. Mater. Exp..

[12] Jutamulia S, Yu F (1996). Overview of hybrid optical neural networks. Opt. Laser Technol..

[13] Fard MMP (2020). Experimental realization of arbitrary activation functions for optical neural networks. Opt. Exp..

[14] Zhou, T. *et al.**Large-scale neuromorphic optoelectronic computing with a reconfigurable diffractive processing unit*. arXiv preprint arXiv:2008.11659 (2020).

[15] Vinckier Q (2015). High-performance photonic reservoir computer based on a coherently driven passive cavity. Optica.

[16] Brunner D, Soriano MC, Mirasso CR, Fischer I (2013). Parallel photonic information processing at gigabyte per second data rates using transient states. Nat. Commun..

[17] Dejonckheere A (2014). All-optical reservoir computer based on saturation of absorption. Opt. Exp..

[18] Nguimdo RM, Verschaffelt G, Danckaert J, Van der Sande G (2014). Fast photonic information processing using semiconductor lasers with delayed optical feedback: Role of phase dynamics. Opt. Exp..

[19] Vandoorne K (2008). Toward optical signal processing using photonic reservoir computing. Opt. Exp..

[20] Sackesyn, S., Ma, C., Dambre, J. & Bienstman, P. An enhanced architecture for silicon photonic reservoir computing. *Proc. Cogn. Comput. 2018: Merg. Concepts with Hardware* (2018).

[21] Laporte F, Katumba A, Dambre J, Bienstman P (2018). Numerical demonstration of neuromorphic computing with photonic crystal cavities. Opt. Exp..

[22] Laporte F, Dambre J, Bienstman P (2019). Highly parallel simulation and optimization of photonic circuits in time and frequency domain based on the deep-learning framework pytorch. Sci. Rep..

[23] Paszke, A. *et al.* Pytorch: An imperative style, high-performance deep learning library. arXiv preprint arXiv:1912.01703 (2019).

[24] Penrose, R. A generalized inverse for matrices. In *Mathematical Proceedings of the Cambridge philosophical Society*, vol. 51, 406–413 (Cambridge University Press, 1955).

[25] Kingma, D. P. & Ba, J. Adam: A method for stochastic optimization. arXiv preprint arXiv:1412.6980 (2014).

[26] http://www.vpiphotonics.com/.

[27] Agrawal, G. P. *Fiber-Optic Communication Systems* Vol. 222 (Wiley, 2012).

[28] Kasper BL, Mizuhara O, Chen Y-K (2002). High bit-rate receivers, transmitters, and electronics. Opt. Fiber Telecommun..

[29] Marcuse D (1991). Calculation of bit-error probability for a lightwave system with optical amplifiers and post-detection gaussian noise. J. Lightwave Technol..

[30] Gray PR (1984). Analysis and Design of Analog Integrated Circuits.

[31] Ip E, Kahn JM (2008). Compensation of dispersion and nonlinear impairments using digital backpropagation. J. Lightwave Technol..

[32] Peddanarappagari KV, Brandt-Pearce M (1997). Volterra series transfer function of single-mode fibers. J. Lightwave Technol..

[33] Cartledge JC, Guiomar FP, Kschischang FR, Liga G, Yankov MP (2017). Digital signal processing for fiber nonlinearities. Opt. Exp..

[34] Anderson C, Lyle J (1994). Technique for evaluating system performance using q in numerical simulations exhibiting intersymbol interference. Electron. Lett..

